# Economic Inequalities in Immunization Coverage Among One-Year-Olds and Coverage Gains from Closing the Inequality Gap in 10 Low- and Middle-Income Countries in the Western Pacific Region, 1994–2021

**DOI:** 10.3390/vaccines13101032

**Published:** 2025-10-03

**Authors:** Ana Mendez-Lopez, Roland Dilipkumar Hensman, Shanlong Ding, Kidong Park

**Affiliations:** Division of Data, Strategy, and Innovation, World Health Organization Regional Office for the Western Pacific, Manila 1000, Philippines; hensmand@who.int (R.D.H.); dings@who.int (S.D.); parkk@who.int (K.P.)

**Keywords:** immunization inequalities, Western Pacific Region, monitoring

## Abstract

Background: Immunization coverage has increased substantially in the Western Pacific Region, saving millions of lives and supporting disease elimination efforts. However, gaps in coverage and inequitable vaccine access persist, leaving millions unvaccinated. Wealth-based inequalities remain a critical barrier to achieving equitable immunization coverage and maximizing the health benefits of vaccination programs. Methods: We analyzed full immunization coverage among 1-year-olds in 10 middle-income countries of the Western Pacific Region using data from the WHO Health Inequalities Data Repository. National and wealth quintile-specific coverage rates and within-country inequalities were assessed using absolute and relative measures (difference, ratio, slope index of inequality, and relative index of inequality). Trends over time were examined in countries with longitudinal data (*n* = 5), identifying pro-rich or pro-poor changes based on shifts in quintile-specific coverage. We also calculated the population attributable risk (PAR) and fraction (PAF) to estimate the potential increase in national coverage if wealth-based inequalities were eliminated. Findings: Substantial gaps in immunization coverage persist across all countries studied (*n* = 10), but with substantial between- and within-country disparities. Coverage was higher among the richest quintiles in half of the countries, with the rest showing no significant disparities. Trends in inequalities were mixed: Cambodia, Mongolia, and Viet Nam experienced pro-poor improvements over time; the Philippines saw widening pro-rich inequalities; and Lao PDR showed little change. Population attributable risks (PAR) showed that eliminating wealth-based inequalities could increase national coverage significantly in five countries (Fiji, Lao PDR, Papua New Guinea, Samoa, and Tonga), with relative gains that could increase national coverage by up to 50% while achieving equity gains. Conclusions: Addressing wealth-based inequalities in immunization could drive substantial gains in national coverage across the Western Pacific Region. Sustained, equity-oriented approaches are essential to achieving universal vaccine access and ensuring no population is left behind. Inequality patterns can guide equity-focused policies to reach underserved and disadvantaged populations.

## 1. Introduction

Since the World Health Organization’s (WHO) Expanded Program on Immunization (EPI) was launched in 1974 with the goal of making vaccines accessible to all, countries worldwide, including those in the Western Pacific Region, have achieved substantial increases in immunization coverage [1,2,3,4]. Immunization services are an essential intervention for reducing child mortality and supporting disease elimination and eradication efforts [5]. In the Western Pacific Region, it is estimated that since the inception of EPI, vaccination has averted approximately 14.5 million deaths, of which 13.6 million averted deaths were among children under 5 years old, contributing to 21% of the reduction in infant mortality in the Region [4].

Vaccine access and immunization programs remain a critical priority and a key component of the primary health care (PHC) approach to health for all [6]. The Immunization Agenda 2030 strategic priority goal 1 calls for effective, efficient, and resilient immunization services to be accessible to all people as an essential part of PHC, contributing to universal health coverage (UHC) [7]. However, despite global and regional gains in immunization levels and continued commitment to vaccine access, substantial coverage gaps persist, leaving millions unvaccinated. Globally, in 2024, coverage with a third dose of the combined diphtheria, tetanus toxoid, and pertussis vaccine (DTP3) was 85% and coverage with a first dose of the measles (MCV1) vaccine was 84% [8]. There were 14.3 million zero-dose children, i.e., children missing out on any vaccination, and 5.6 million children only partially vaccinated [8]. Additionally, very low immunization coverage levels persisted for some vaccines, such as for the human papillomavirus (HPV), which globally stood at 31% 2024, and the yellow vaccine fever in countries at risk, for which coverage in 2024 was just at 50%, well below the recommended level of 80% [8]. There were large disparities in coverage among countries of varying income levels, with most of the people missing out on vaccines living in low-resource settings, highlighting the global disparities between countries in vaccine access [8].

These coverage challenges were exacerbated by the COVID-19 pandemic, which further hindered efforts to ensure equitable vaccine access [9,10,11]. Nearly all countries reported disruptions in vaccine coverage due to pandemic-related disruptions, including 95% of countries in the East Asia and the Western Pacific Region [10,12]. Globally, immunization with DTP3 dropped from 86% in the pre-pandemic year 2019 to 82% in 2021, later reaching 85% in 2024 [8,13]. A similar trend was observed for MCV1 coverage. Although some recovery began in 2022, immunization coverage in most countries has yet to return to pre-pandemic levels.

Equity in immunization is a key guiding principle of the post-pandemic program of work of the strategic coordinated efforts by WHO, United Nations Children’s Fund (UNICEF), and Gavi, the Vaccine Alliance under The Big Catch-up, an essential immunization recovery plan for 2023 and beyond [14]. The plan aims to catch up on missed vaccinations, restore vaccination coverage rates to at least pre-pandemic levels, and strengthen immunization systems through PHC approaches. The Big Catch-up equity-oriented plan focuses efforts on the most vulnerable populations, areas and communities with the most missed children or those facing persistent or acute barriers to vaccination services. This global initiative recognizes that the backsliding of immunization rates during COVID-19 has created a large immunity gap that must be closed urgently. It emphasizes three approaches—catch-up, restore, and strengthen—to not only deliver missed vaccines but also rebuild resilient immunization delivery as part of primary care. By concentrating on underserved communities, The Big Catch-up seeks to ensure that immunization recovery is inclusive and leaves no one behind.

Wealth-based inequalities are a critical barrier to achieving equitable health outcomes, including immunization coverage and maximizing the health benefits of vaccination programs [15,16,17]. Inequalities in immunization coverage are particularly resilient in low- and middle-income countries where socioeconomic disparities are often more pronounced, resources are limited, and health systems may be less resilient to shocks such as from the COVID-19 pandemic [18]. These inequalities not only leave the most disadvantaged children unvaccinated but also undermine broader public health efforts by perpetuating disease transmission and limiting the overall impact of immunization programs. Investigating wealth-based inequalities is essential for identifying the populations most at risk of being left behind and developing targeted strategies to close coverage gaps, ensuring that immunization services leave no one behind, ultimately contributing to achieving UHC and reducing child mortality [19]. Equity analyses provide the evidence to drive action: by pinpointing which communities or subgroups have low coverage, policymakers and partners can tailor interventions (such as outreach clinics, community engagement, or fee removal) to address the specific barriers in those populations.

Here, we analyze wealth-based inequalities and immunization coverage gaps in 10 middle-income countries of the Western Pacific Region. We assess the latest status and trends of national and wealth quintile-specific coverage rates and within-country inequalities. We also investigate the potential impact on national coverage levels if wealth-based inequalities were eliminated to identify where policies targeted to the poorest and economically disadvantaged populations could improve equitable access to immunization services. By quantifying how much coverage could increase through equity-focused efforts, our analysis provides evidence to inform regional immunization strategies and underscore the importance of addressing inequalities as part of immunization programs.

## 2. Methods

### 2.1. Data Source and Country Selection

Data were sourced from the WHO Health Inequalities Repository, which provides data for multiple health indicators disaggregated by several dimensions of inequality [20,21]. Estimates for this study for countries in the WHO Western Pacific Region are produced based on a reanalysis of household survey micro-level data from Demographic and Health Surveys and Multiple Indicator Cluster Surveys [22,23].

We included in the analysis all ten countries from the WHO Western Pacific Region with available data with at least one data point after 2015, and complete observations for all subgroups. The countries meeting these criteria were: Cambodia, Fiji, Kiribati, Lao People’s Democratic Republic (Lao PDR), Mongolia, Papua New Guinea, the Philippines, Samoa, Tonga, and Viet Nam (Table S1).

### 2.2. Immunization Measurement

Immunization coverage was measured as the percentage of one-year-olds who had received one dose of the Bacille Calmette-Guérin vaccine (BCG), three doses of the polio vaccine (PV3), three doses of the combined diphtheria, tetanus toxoid, and pertussis vaccine (DTP3), and one dose of measles vaccine (MCV1). These vaccines were selected as they are part of the WHO-recommended core set for monitoring routine childhood immunization coverage globally, as well as based on data availability in the WHO Health Inequalities Repository. This corresponds to a standard definition of full basic childhood immunization used in many coverage surveys. In other words, a child was considered “fully immunized” for the analysis if they received all of the above doses by the age of one.

### 2.3. Measurement of Economic Inequalities

Economic inequalities were assessed using a wealth index as provided in the WHO Health Inequalities Repository. Country-specific indices were constructed using principal component analysis based on owning selected assets and having access to certain basic services (e.g., water, sanitation, electricity), and do not directly include access to health services. Within each country, the index was divided into five equal subgroups, with each wealth quintile accounting for 20% of the population. This asset-based wealth quintile approach is a standard proxy for socioeconomic status in DHS and MICS analyses. It captures relative wealth within a country, which is useful for assessing inequality, though it may not capture all aspects of poverty.

### 2.4. Statistical Analysis

#### 2.4.1. Summary Statistics and Patterns of Coverage

We calculated summary statistics and inequality measures for each country’s latest immunization data, stratified by wealth quintile. We calculated mean immunization coverage across all countries and for the poorest and richest quintiles separately. The coverage gap was defined as the difference between universal coverage (100%) and each country’s actual coverage. We assessed inequality patterns across countries as linear (even gaps between quintiles), bottom (poorest quintiles lagging), or top (wealthiest quintile ahead of the others).

#### 2.4.2. Inequality Measures

We then calculated simple and complex inequality measures to evaluate their level and impact on progress toward universal immunization coverage. Simple summary measures included the difference (D), a measure of absolute inequality calculated as the difference in coverage between the richest and poorest wealth quintiles, and the ratio (R), a measure of relative inequality calculated by dividing the coverage in the poorest wealth quintiles by the coverage in the richest wealth quintile [24,25].

Complex measures, the slope index of inequality (SII) and relative index of inequality (RII), use regression models to estimate differences and ratios between the richest and poorest quintiles, accounting for all quintile estimates and weights [24,25]. We compared SII values with national coverage to assess whether wealth-based inequalities persisted in high-coverage contexts or diminished as national immunization increased.

#### 2.4.3. Longitudinal Analysis of Inequality Trends

To analyze changes in wealth-based inequalities over time, we first calculated SII for all available data in countries with at least two distinct years. Second, we examined how changes in inequality patterns related to shifts in national immunization coverage to explore whether equity gains coincided with overall coverage increases. We compared each country’s most recent data with a previous data point at least five years apart (preferably closest to 2015 or the nearest alternative).

We calculated trends in inequality patterns as the annual absolute excess change in the poorest quintile compared with the richest quintile against the annual absolute change in the national average. For each quintile, we estimated the annual pace of change by dividing the coverage difference between the latest and earlier data points by the number of years separating them. We then subtracted the richest quintile’s annual change from that of the poorest, obtaining the annual excess change (in percentage points). A positive excess change indicates a pro-poor trend, where coverage grew faster in the poorest quintile. A negative value indicates a pro-rich trend, with faster gains in the richest quintile [15,25].

#### 2.4.4. Impact Measures of Reducing Inequalities

Finally, we calculated additional complex measures to assess the impact of addressing wealth-based inequalities. These quantified potential improvements in national coverage if all quintiles matched the wealthiest quintile. Impact measures included the population attributable risk (PAR), an absolute inequality metric defined as the difference between the wealthiest quintile’s coverage and the national average [24,25]. PAR reflects the potential increase in immunization coverage (in percentage points) if the gap between the wealthiest quintile and the rest were closed. We also calculated the population attributable fraction (PAF), a relative measure derived by dividing PAR by the national average and multiplying by 100. PAF indicates the percentage improvement in coverage achievable by closing the gap between the richest quintile and the rest.

Data were analyzed using Stata/MP 17, including the *healthequal* module [26,27]. Regional overall values were weighted by the country- and quintile-specific populations.

## 3. Results

### 3.1. Coverage Levels

Full immunization coverage across the 10 countries for the latest year (2017–2022) was 58.4.3% (95%CI: 41.9–74.9), equivalent to an average coverage gap of 39.7% of the population of one-year-olds not having received the full course of vaccinations for BCG-PV3-DTP3-MCV1 (Table 1). Coverage ranged from the lowest coverage in Kiribati at 13.4 to the highest in Fiji at 89.0%. The proportion of vaccination data obtained from records versus maternal recall varied across countries, ranging from 71.0% (Lao PDR) to 99.9% (Tonga) of children with vaccination information based on records, with the remaining based on maternal recall (Table S2).

### 3.2. Patterns of Inequality

**Figure 1:**
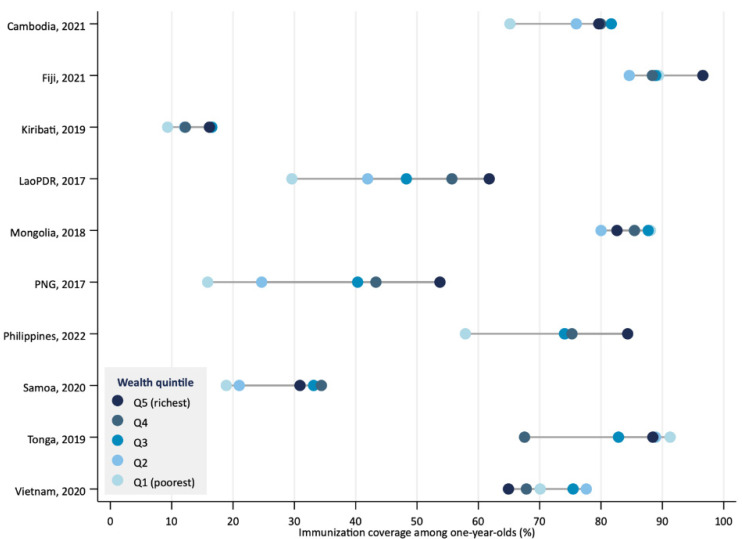
Coverage of full immunization by wealth quintile in countries of the Western Pacific Region, latest year.

Countries showed different patterns of inequalities in immunization coverage, including linear, bottom, and top disparities. Lao PDR and Papua New Guinea exhibited linear patterns, with similar gaps between quintiles and a roughly dose–response relationship, where higher wealth levels correlated with increased coverage (Figure 1). Cambodia and Samoa displayed a bottom pattern, with the poorest quintiles having lower coverage than all others, meaning the poorest are left behind. Fiji showed a top pattern, with the wealthiest quintile having higher coverage than all others. Some countries had no clear pattern, with inconsistent coverage levels across different wealth groups.

### 3.3. Levels of Inequality

Across countries, the average coverage of full immunization was higher in the richest wealth quintile (65.9%, 95%CI: 49.3–82.5) compared to the poorest (53.6%, 95%CI: 32.9–74.2), a non significant difference of 12.4 percentage points (95%CI: −13.4–38.2) and ratio of 1.2 (95%CI: 0.8–1.9) in favor of the richest quintile (Table 1).

Within countries, in half of them (Cambodia, Lao PDR, Papua New Guinea, Philippines, and Samoa), coverage was higher among the richest quintile (Table 1). In two countries (Kiribati and Fiji), this difference was not significant. In three countries (Mongolia, Tonga, and Viet Nam), coverage was higher among the poorest, but there was no significant difference.

Inequalities between the richest and poorest varied substantially across countries. The largest difference was observed in Papua New Guinea, where coverage among the richest was 3.4 (95%CI: 2.4–4.8) times higher than among the poorest, with coverage in the richest group being 37.9 (95%CI: 30.3–45.5) percentage points higher (Table 1). The countries that followed were Lao PDR and the Philippines, where the gaps in coverage between the poorest and the richest were 32.2 percentage points (95%CI: 24.8–39.5) and 26.5 percentage points (95%CI: 15.9–37.0), respectively.

When assessing coverage across all quintiles to measure inequalities, the SII and RII produced results similar to the difference and ratio measures, except that in Kiribati, inequalities were statistically significant when using these more complex measures (unlike when measured with the simple difference and ratio measures) (Table 1).

**Figure 2:**
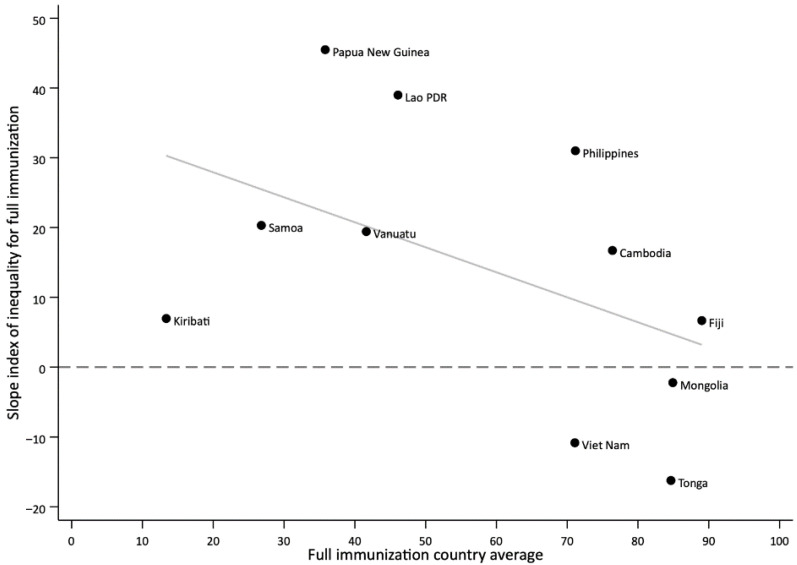
Relationship between national average and wealth-based inequalities in immunization coverage as measured with the slope index of inequality (SII) in countries of the Western Pacific Regions, latest year.

Countries with lower average coverage often had higher levels of wealth-based inequality in full immunization, as measured by the SII (Figure 2). For example, Papua New Guinea and Lao PDR had low coverage and high SII values. Conversely, Viet Nam and Tonga had high coverage and negative SII values, indicating no disparities or slightly higher coverage among poorer quintiles. However, this relationship was not consistent across all countries. The Philippines and Viet Nam had similar national coverage but differed substantially in inequalities, with the Philippines showing a positive SII and Viet Nam a negative one.

### 3.4. Inequality Trends

**Figure 3:**
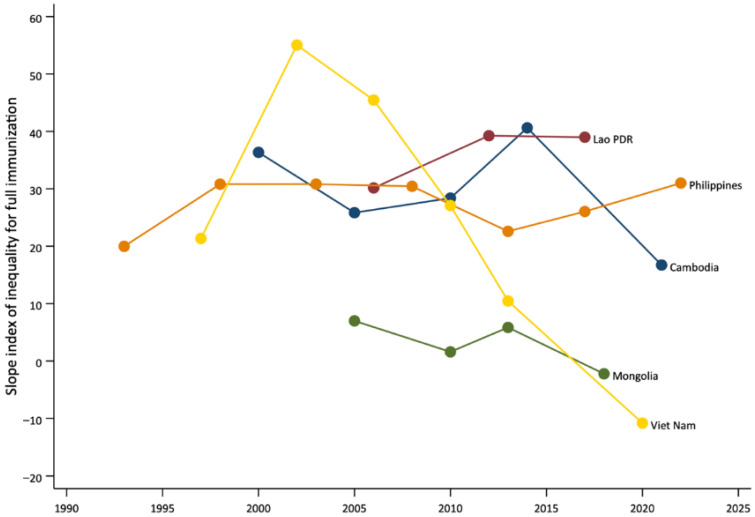
Trends in inequalities in coverage of full immunization by wealth quintile in countries of the Western Pacific Region, change over time.

In countries with longitudinal data (*n* = 5), trends in wealth-based inequalities in full immunization coverage, as measured by the SII, varied over time (Figure 3). Lao PDR and Philippines showed persistently high levels of inequality across survey years, with increased inequality gaps over time. In Cambodia and Mongolia, inequalities initially fluctuated but decreased in the latest survey. By contrast, Viet Nam showed marked reductions in inequality over time. In the latest survey, no wealth-based inequalities were observed in Mongolia and Viet Nam.

**Figure 4:**
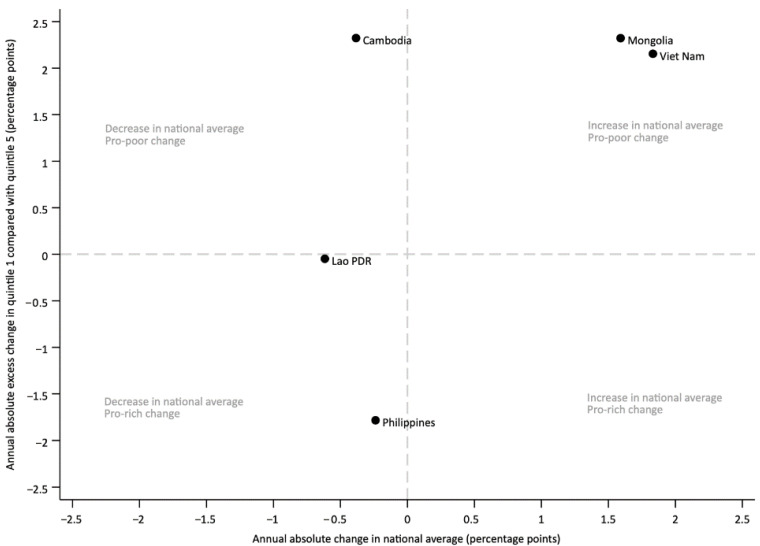
Joint change over time in national average immunization coverage among one-year-olds, and in the richest quintile compared to the poorest.

Joint changes in national average coverage and absolute inequalities between the most recent survey and the preceding one (separated by at least five years) further illustrate these patterns (Figure 4). Mongolia and Viet Nam experienced increases in national average coverage accompanied by pro-poor improvements, reducing disparities between the richest and poorest quintiles. Cambodia also showed pro-poor changes despite a slight decline in national average coverage. In contrast, Lao PDR experienced reduced coverage with minimal change in inequalities, and the Philippines saw small declines in national coverage along with widening inequalities.

### 3.5. Impact of Eliminating Wealth-Based Inequalities on the National Coverage Level

**Figure 5:**
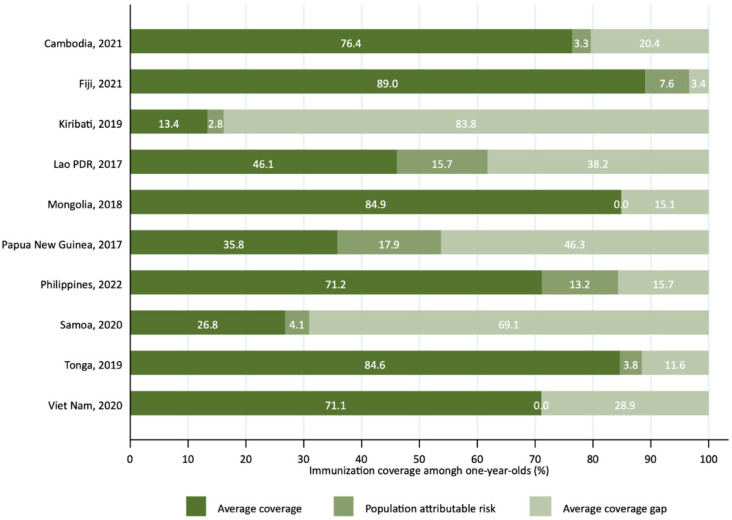
Population attributable risk for the potential reduction in the national immunization coverage gap by eliminating wealth-based inequalities in countries of the Western Pacific Region, latest year.

PAR results showed that in half of the countries (*n* = 5), substantial gains in national immunization coverage could be achieved by eliminating the differences in coverage between the richest quintile and the rest. This means that if all wealth quintiles reached the same coverage level as the wealthiest, national coverage could increase significantly. The largest potential improvements were observed in Papua New Guinea (17.9 percentage points, 95%CI: 13.2–22.6), Lao PDR (15.7 percentage points, 95%CI: 11.1–20.3), and Philippines (13.2 percentage points, 95%CI: 8.7–17.7), where national immunization coverage rates could increase by up to 17.9 percentage points if inequalities between wealth quintiles were eliminated (Table 1 and Figure 5). The PAF further illustrates this potential by showing relative gains: eliminating wealth-based inequalities could increase national coverage by 50% in Papua New Guinea (95%CI: 49.9–50.2), 34% in Lao PDR (95%CI: 33.9–34.1), and 18.5% in Philippines (95%CI: 18.5–18.6) (Table 1).

## 4. Discussion

This study highlights persistent wealth-based inequalities in full immunization coverage across 10 middle-income countries in the WHO Western Pacific Region. While average national coverage has improved in some settings, disparities between the richest and poorest households remain a critical barrier to achieving equitable immunization. In half of the countries analyzed, children from the wealthiest quintiles had higher coverage rates than those from the poorest, with particularly large disparities in Papua New Guinea, Lao PDR, and the Philippines. Conversely, Mongolia and Viet Nam demonstrated relatively equitable coverage, with no significant differences between wealth groups in the most recent surveys.

These findings align with the equity ambitions of the Immunization Agenda 2030, which emphasizes the need to leave no one behind by reducing inequalities in immunization access and ensuring that every person benefits from vaccines throughout their life course. Despite substantial regional gains since the inception of the EPI, equity gaps remain in the Western Pacific Region, where progress is often uneven across and within countries. Wealth-related disparities, compounded by geographic and systemic barriers, continue to hinder universal and equitable access to health services, including immunization, a key component of the Region’s strategy to strengthen PHC and advance UHC [28]. Our results reinforce that achieving high national immunization coverage alone is not sufficient for UHC or disease elimination goals if certain subgroups are left with low coverage.

In all study countries, the vaccines analyzed in this paper (BCG, PV3, DTP3, MCV1) are provided free of charge through government immunization programs, with several countries having legislation that is supportive of immunization and commits the government to finance all aspects of the immunization program at all levels [29,30]. Private providers may charge a service fee, though contribution is mostly limited to less than 10% of the total target population [31]. Nonetheless, coverage disparities may persist due to health system limitations, including geographic accessibility, cold chain infrastructure, and workforce availability, which can impede equitable uptake even in the absence of direct costs.

Differences in vaccination coverage between countries may also be partly influenced by the implementation of campaigns and other activities to promote demand for vaccines. In recent years, countries in the Western Pacific Region have undertaken efforts such as measles and polio campaigns, community engagement initiatives, public communication strategies, and service quality improvements to address under-vaccination [32]. Several of these activities likely contributed to narrowing gaps or boosting uptake in the short term, which may partly explain some of the variation in coverage patterns observed across surveys.

Our results also reflect the broader challenges highlighted in the WHO Western Pacific Region’s Strategic Framework for Vaccine-Preventable Diseases and Immunization in the Western Pacific 2021–2030, which calls for resilient immunization systems that prioritize equity and are responsive to the needs of marginalized populations [33]. The COVID-19 pandemic further disrupted routine immunization services and widened inequities, particularly affecting children in lower socioeconomic quintiles [9,10]. Recovery efforts, such as The Big Catch-up initiative launched by WHO, UNICEF, and Gavi, focus on restoring immunization coverage to pre-pandemic levels and accelerating progress by targeting underserved areas and populations [14].The patterns of inequality observed in our study suggest that such efforts will need to be both equity-oriented and context-specific to be effective.

Longitudinal analyses revealed mixed trends. In Viet Nam and Mongolia, marked reductions in inequality over time were accompanied by increases in national coverage, illustrating the potential for pro-poor progress when equitable strategies are implemented. By contrast, in Lao PDR and the Philippines, inequalities widened or remained unchanged, and coverage gains were either minimal or reversed. These divergent trends underscore that improving national coverage alone does not guarantee equitable outcomes. Additionally, the overall negative slope in Figure 2 highlights a general tendency for inequalities to narrow as national coverage increases, but exceptions underscore the importance of examining both average coverage and equality measures. In a few cases, coverage appeared higher among poorer quintiles, suggesting counterintuitive “pro-poor” patterns. However, in most of these instances, the confidence intervals overlapped, indicating that differences may not be statistically meaningful. Such results could also reflect targeted outreach or campaign activities that prioritize disadvantaged populations, as well as variation due to survey timing or sampling. While these findings highlight the potential for equity-focused strategies to achieve pro-poor progress, they should be interpreted with caution. These findings reinforce the need for equity-focused strategies that address gaps among disadvantaged groups alongside efforts to improve overall immunization levels. This could include setting specific equity targets in national plans, as well as employing innovative solutions like integration of immunization with PHC, cross-subsidization, community-based tracking, and engaging non-health sectors to reach the poorest families [34,35,36].

Addressing wealth-based disparities could substantially increase national coverage in some settings. The PAR results suggest that eliminating these inequalities could increase national immunization coverage by nearly 18 percentage points in Papua New Guinea and over 15 percentage points in Lao PDR, resulting in relative gains of 50% and 34%, respectively. Such improvements would accelerate progress toward the Immunization Agenda 2030 targets and make a meaningful contribution to child survival and health equity. This is especially relevant in countries where large birth cohorts and high levels of inequality coexist, amplifying the potential health gains of equity-focused policies. Our analysis, therefore, provides a quantitative rationale for policymakers and donors to invest in equity-oriented interventions, not only as a matter of social justice but as a highly effective strategy to increase coverage and improve health.

Several limitations must be acknowledged. First, the analysis included less than half of the countries in the Region that had available disaggregated data post-2015, and, therefore, the findings may not be generalizable to countries without recent or high-quality data. Second, household surveys, such as the DHS and MICS, are subject to recall bias and may underrepresent hard-to-reach populations, potentially underestimating true inequalities. Relatedly, the proportion of vaccination data based on records versus caregiver recall varied across countries, which may affect the comparability and robustness of estimates. These differences in data sources highlight the importance of cautious interpretation, as reliance on recall may introduce additional bias in some settings. Third, differences in survey design and implementation may limit the comparability of results across countries and time. Finally, the use of wealth quintiles captures relative economic position within countries but may not fully reflect multidimensional poverty or intersecting inequities (e.g., by geography, ethnicity, or maternal education). Importantly, the DHS and MICS wealth index is constructed from household assets, dwelling characteristics, and access to basic services, and does not directly include access to health services, reducing the risk of circularity in the findings. Further studies should investigate immunization inequalities by other dimensions, as well as how these factors intersect with each other. Of particular relevance to the Western Pacific Region are geographic barriers, especially in Pacific island countries, where populations are dispersed across remote outer islands. In such settings, supply-side challenges—including limited cold-chain capacity, irregular transport links, and constrained health workforce availability—can make timely vaccine delivery difficult and dependent on infrequent outreach visits [37,38].

Despite these limitations, this study offers valuable insights for informing policy and programmatic action. Countries with high pro-rich disparities, such as Papua New Guinea and Lao PDR, may benefit from targeted interventions focusing on the poorest quintiles. Strategies could include strengthening outreach services in underserved areas, removing financial and non-financial barriers to access, and deploying community health workers to extend immunization services beyond fixed facilities. Countries like Mongolia and Viet Nam, which achieved equity gains, may offer valuable lessons on implementing integrated PHC approaches that effectively reach disadvantaged groups.

Regular monitoring of immunization coverage, disaggregated by socioeconomic status, is essential to track progress and guide effective interventions. Equity-oriented planning and implementation, informed by disaggregated data, will be critical to ensuring that immunization systems leave no one behind and contribute fully to UHC and the SDGs. Strengthening health information systems to produce timely and reliable disaggregated data, as recommended by Immunization Agenda 2030 and regional strategies, will be crucial to sustaining progress and preventing the resurgence of inequities.

## 5. Conclusions

Reducing wealth-based inequalities in immunization coverage is vital to achieving universal and equitable access to vaccines in the Western Pacific Region. Our findings underscore the need for equity-oriented strategies that simultaneously improve national coverage and prioritize disadvantaged populations. Targeted and context-specific interventions, combined with robust monitoring systems, will be essential for countries to realize the equity vision of Immunization Agenda 2030 and strengthen resilience against future health system shocks.

## Figures and Tables

**Table 1:** Coverage of full immunization by wealth quintiles and inequality measures in the Western Pacific Region, latest year.

Setting	Year	Setting Average	Poorest Quintile (Q1)	Richest Quintile (Q5)	Difference (D)	Ratio (R)	Slope Index of Inequality (SII)	Relative Index of Inequality (RII)	Population Attributable Risk (PAR)	Population Attributable Fraction (PAF)
Overall (mean)	2017–2021	58.4	53.6	65.9	12.4	1.2	14.2	1.3	5.9	9.8
		(41.9 to 74.9)	(32.9 to 74.2)	(49.3 to 82.5)	(−13.4 to 38.2)	(0.8 to 1.9)	(10.9 to 17.6)	(1.2 to 1.3)	(4.0 to 7.8)	(9.8 to 9.8)
Cambodia	2021	76.4	65.2	79.6	14.5	1.2	16.7	1.2	3.3	4.3
			(58.7 to 71.1)	(72.8 to 85.1)	(5.8 to 23.2)	(1.1 to 1.4)	(4.5 to 29.0)	(1.1 to 1.5)	(−0.6 to 7.1)	(4.2 to 4.3)
Fiji	2021	89.0	89.4	96.6	7.2	1.1	6.7	1.1	7.6	8.5
			(82.2 to 93.9)	(87.5 to 99.1)	(−0.1 to 14.5)	(1.0 to 1.2)	(−5.8 to 19.1)	(0.9 to 1.2)	(2.5 to 12.7)	(8.5 to 8.6)
Kiribati	2019	13.4	9.3	16.2	6.8	1.7	7.0	1.7	2.8	20.9
			(4.4 to 18.7)	(9.2 to 26.9)	(−4.2 to 17.8)	(0.7 to 4.3)	(3.0 to 10.9)	(1.3 to 2.3)	(−3.7 to 9.3)	(20.4 to 21.4)
Lao PDR	2017	46.1	29.6	61.8	32.2	2.1	39.0	2.4	15.7	34.0
			(25.6 to 34.0)	(55.6 to 67.6)	(24.8 to 39.5)	(1.8 to 2.5)	(34.6 to 43.4)	(2.1 to 2.8)	(11.1 to 20.3)	(33.9 to 34.1)
Mongolia	2018	84.9	88.1	82.6	−5.5	0.9	−2.2	1.0	0.0	0.0
			(82.4 to 92.1)	(69.5 to 90.8)	(−17.0 to 6.1)	(0.8 to 1.1)	(−10.6 to 6.2)	(0.9 to 1.1)	(−4.7 to 4.7)	(−0.1 to 0.1)
Papua New Guinea	2017	35.8	15.9	53.7	37.9	3.4	45.5	4.0	17.9	50.0
			(11.2 to 22.0)	(48.4 to 59.0)	(30.3 to 45.5)	(2.4 to 4.8)	(39.4 to 51.6)	(3.1 to 5.1)	(13.2 to 22.6)	(49.9 to 50.2)
Philippines	2022	71.2	57.9	84.3	26.5	1.5	31.0	1.6	13.2	18.5
			(51.6 to 63.9)	(73.9 to 91.1)	(15.9 to 37.0)	(1.3 to 1.7)	(23.4 to 38.7)	(1.4 to 1.8)	(8.7 to 17.7)	(18.5 to 18.6)
Samoa	2020	26.8	18.9	30.9	12.0	1.6	20.3	2.2	4.1	15.4
			(12.8 to 27.1)	(22.8 to 40.5)	(0.7 to 23.4)	(1.0 to 2.6)	(10.6 to 30.1)	(1.5 to 3.0)	(−4.7 to 12.9)	(15.1 to 15.7)
Tonga	2019	84.6	91.3	88.4	−2.8	1.0	−16.2	0.8	3.8	4.5
			(77.0 to 97.0)	(74.3 to 95.3)	(−16.1 to 10.5)	(0.8 to 1.1)	(−43.2 to 10.8)	(0.6 to 1.2)	(5.7 to 13.3)	(4.4 to 4.6)
Viet Nam	2020	71.1	70.1	64.9	−5.2	0.9	−10.8	0.9	0.0	0.0
			(61.9 to 77.2)	(55.0 to 73.6)	(−17.3 to 7.0)	(0.8 to 1.1)	(−23.8 to 2.2)	(0.7 to 1.0)	(−5.8 to 5.8)	(−0.1 to 0.1)

Note: 95% confidence interval in brackets.

## Data Availability

The data used are published in the WHO Health Inequality Data Repository at https://www.who.int/data/inequality-monitor/data (accessed on 23 July 2024).

## Supplementary Materials

The following supporting information can be downloaded at: https://www.mdpi.com/article/10.3390/vaccines13101032/s1, Table S1: List of countries, surveys, and years of data availability; Table S2: Proportion of vaccination coverage information sources among children aged 12–23 months, crude coverage for selected basic antigens.
