# Supplementary material for: Economic Inequalities in Immunization Coverage Among One-Year-Olds and Coverage Gains from Closing the Inequality Gap in 10 Low- and Middle-Income Countries in the Western Pacific Region, 1994–2021
Source: Vaccines (Basel). 2025 Oct 3;13(10):1032. doi: 10.3390/vaccines13101032 (PMC12567940; doi:10.3390/vaccines13101032)
Supplement: Supplementary file 1 [file vaccines-13-01032-s001.zip › vaccines-3814354-supplementary.pdf]

Online supplementary material

Table S1. List of countries, surveys, and years of data availability.

Country	Year	Source
Cambodia	2000	DHS
	2005	DHS
	2010	DHS
	2014	DHS
	2021	DHS
Fiji	2021	MICS
Kiribati	2019	MICS
Lao People's Democratic Republic	2006	MICS
	2012	DHS/MICS
	2017	MICS
Mongolia	2005	MICS
	2010	MICS
	2013	MICS
	2018	MICS
Papua New Guinea	2017	DHS
Philippines	1993	DHS
	1998	DHS
	2003	DHS
	2008	DHS
	2013	DHS
	2017	DHS
	2022	DHS
Samoa	2020	DHS/MICS
Tonga	2019	MICS
Viet Nam	1997	DHS
	2002	DHS
	2006	MICS
	2010	MICS
	2013	MICS
	2020	MICS

Notes: DHS: Demographic and Health Survey; MICS: Multiple Indicator Cluster Survey.

Table S2. Proportion of vaccination coverage information sources among children aged 12–23 months, crude coverage for selected basic antigens.

Country	Survey	Year	Vaccination records	Mother's report	Either	Vaccination records	Mother's report
			All children aged 12-23 months old			Vaccinated children aged 12-23 months old	
Cambodia	DHS	2021	66.9	9.5	76.4	87.6	12.4
Fiji	MICS	2021	92	3.4	95.4	96.4	3.6
Kiribati	MICS	2019	18.6	4.7	23.4	79.5	20.1
Lao PDR	MICS	2017	34.6	13.5	48.1	71.9	28.1
Mongolia	MICS	2018	84	2.9	86.9	96.7	3.3
Philippines	DHS	2022	59.7	12.1	71.8	83.1	16.9
Samoa	MICS	2020	26.6	0.1	26.8	99.3	0.4
Tonga	MICS	2019	93.4	0.2	93.5	99.9	0.2
Viet Nam	MICS	2020	60.7	0.7	61.3	99.0	1.1
